# Predictors of chronic kidney disease in type 2 diabetes: A longitudinal study from the AMD Annals initiative: Erratum

**DOI:** 10.1097/MD.0000000000005660

**Published:** 2016-12-09

In the article “Predictors of chronic kidney disease in type 2 diabetes: A longitudinal study from the AMD Annals initiative”,^[1]^ which appeared in Volume 95, Issue 27 of *Medicine*, Dr. Ceriello's second affiliation was originally omitted. Dr. Ceriello is affiliated with the Department of Cardiovascular and Metabolic Diseases, IRCCS Gruppo Multimedica, Sesto San Giovanni, MI, Italy as well as the Institut d’Investigacions Biomèdiques August Pii Sunyer (IDIBAPS) and Centro de Investigación Biomédicaen Red de Diabetes y Enfermedades Metabólicas Asociadas (CIBERDEM), Barcelona, Spain.
